# Native-valve endocarditis detected by point-of-care echocardiography

**DOI:** 10.1186/s13089-022-00294-2

**Published:** 2022-10-27

**Authors:** Pablo Blanco, Liliana Figueroa, María Fernanda Menéndez

**Affiliations:** High-Dependency Unit (UCIM), Hospital “Dr. Emilio Ferreyra”, 4801, 59 St., 7630 Necochea, Argentina

**Keywords:** Infective endocarditis, Echocardiogram, Point-of-care

## Abstract

**Background:**

Infective endocarditis carries a high morbidity and mortality; therefore, a rapid diagnosis and timely treatment is crucial to improve outcomes. Diagnosis of infective endocarditis is supported on echocardiogram findings.

**Case presentation:**

An adult male with history of long-term hemodialysis, presented with embolic manifestations (cerebral, skin) and fever. A large vegetation in the mitral valve and other in the tricuspid valve were detected by point-of-care transthoracic echocardiogram, while blood cultures subsequently resulted positive for methicillin-resistant *Staphylococcus aureus.* Despite therapeutic efforts, the patient developed into an irreversible shock and died.

**Conclusions:**

Point-of-care echocardiogram has a pivotal role in diagnosis and decision-making of infective endocarditis.

**Supplementary Information:**

The online version contains supplementary material available at 10.1186/s13089-022-00294-2.

## Background

Infective endocarditis (IE) carries a high morbidity and mortality that are mostly related to heart failure and embolism. Therefore, reaching an early diagnosis is essential for guiding appropriate and timely interventions.

IE alludes to the infection of the endocardium. It typically involves the valves and/or perivalvular tissue but can also include the subvalvular apparatus, mural endocardium, ascending aorta, Eustachian valve or Chiari network, and electronic intracardiac devices (e.g., pacemaker leads). IE is broadly classified in native-valve endocarditis and prosthetic-valve endocarditis.

Here, we presented a case of an adult male with a typical case of native-valve endocarditis where point-of-care (POCUS) transthoracic echocardiogram (TTE) aided in establishing the diagnosis and prognosis. Then, we succinctly review the disease and the role of POCUS on diagnosis and decision-making.

## Case presentation

**Fig. 1:**
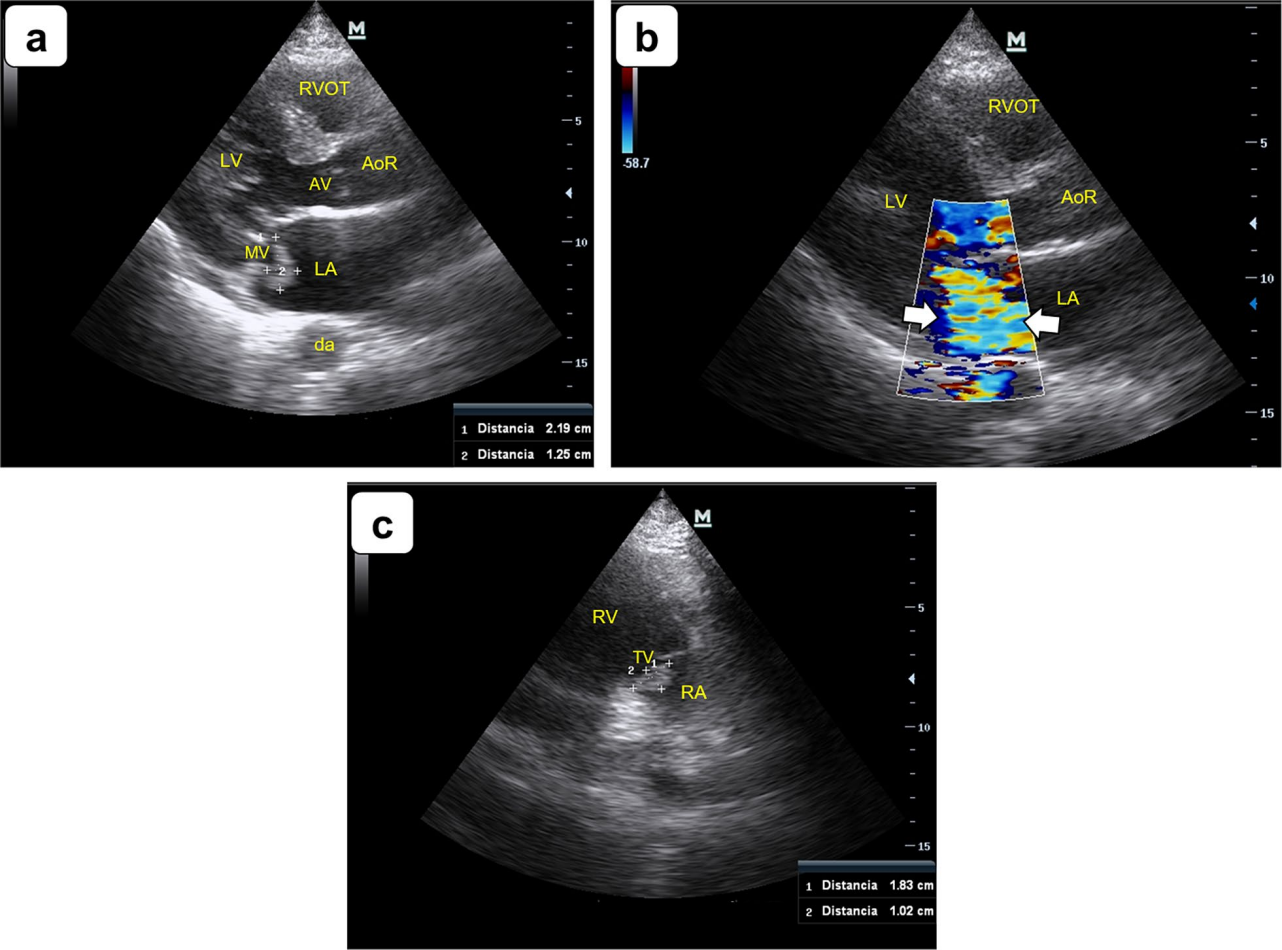
Point-of-care transthoracic echocardiogram showing signs of infective endocarditis. **a** Parasternal long axis (PLAX) view depicting a vegetation measuring 22 mm × 13 mm (calipers) attached to the atrial side of the anterior leaflet of the mitral valve. **b** PLAX showing a severe mitral regurgitation (arrows) triggered by the mitral valve vegetation. **c** Right ventricular inflow view showing a vegetation of 18 mm × 10 mm (calipers) in the atrial side of the posterior leaflet of the tricuspid valve. *LV* left ventricle, *LA* left atrium, *AoR* aortic root, *AV* aortic valve, *MV* mitral valve, *da* descending aorta, *RVOT* right ventricular outflow tract, *RV* right ventricle, *RA* right atrium, *TV* tricuspid valve

A 43-year-old male was brought to the Emergency Department (ED) with a left-sided hemiparesis and mixed aphasia. He had a long-term history of end-stage renal failure on hemodialysis with difficult vascular accesses because of infection-related devices (two failed prosthetic arteriovenous grafts in the upper arms, loss of a cuffed catheter in the left subclavian vein and several losses of non-tunneled catheters). On presentation, vital signs were a heart rate of 110 beats/min., respiratory rate of 20 breaths/min., axillary temperature of 38 °C (100.4°F), blood pressure of 160/90 mmHg and pulse oxygen saturation of 96% breathing on room air. Glasgow Coma Scale was 12/15 (M: 5; E: 3; V: 4), with mixed aphasia and left-sided hemiparesis. Several petechiae were found in conjunctivae and in the hands and feet. The patient had a non-cuffed hemodialysis catheter placed in the right femoral vein that was used for renal replacement therapy. Relevant data on blood analysis were a hemoglobin level of 7 g/dL, platelet count of 16,000 per microliter, a prothrombin time of 18 s and a fibrinogen level of 130 mg/dL (overt disseminated intravascular coagulation). Two sets of blood cultures were taken at admission. Cranial computed tomography showed a right-sided parietal and occipital lobe infarcts. A point-of-care transthoracic echocardiogram (TTE) showed a non-dilated left ventricle with moderate impairment of the systolic function. A pedunculated oscillating mass measuring 22 mm × 13 mm was attached to the atrial side of the anterior leaflet of the mitral valve which triggered a severe mitral regurgitation (Fig. 1a and b and Additional file 1: Video S1 and Additional file 2: Video S2). Also, a fixed mass measuring 18 mm × 10 mm was observed attached to the atrial side of the posterior leaflet of the tricuspid valve; minor tricuspid regurgitation was noted (Fig. 1c and Additional file 3: Video S3). Aortic and pulmonary valve leaflets were thin, with no stenosis or regurgitation. Left ventricle filling pressure was elevated. Size and systolic function of the right ventricle were normal. Based on the whole clinical picture and POCUS-TTE findings, the diagnosis of infective endocarditis (IE) was first considered. He started on vancomycin and meropenem. Subsequently, methicillin-resistant Staphylococcus *aureus* grown in blood cultures and IE was therefore confirmed. Given the lack of cardiovascular surgery capabilities in the hospital and that the patient rapidly developed into an irreversible shock, he died shortly thereafter.

## Discussion

The diagnosis of IE can be reached using validated criteria which are mainly based on blood culture results and echocardiographic findings, and several minor criteria [1–3]. Recently, 18F-fluorodeoxyglucose positron emission tomography/computed tomography (18F-FDG PET/CT) showed also a role in diagnosing prosthetic-valve endocarditis [4].

Vegetations (i. e., oscillating masses on the low-pressure side of valvular leaflets), perivalvular abscess (i.e., anechoic area in the perivalvular tissue, which if filled on color Doppler is indication of a pseudoaneurysm) and a new dehiscence of a prosthetic valve (typically detected as a pathologic perivalvular leak) are the tree main echocardiographic findings of IE [1]. Minor echocardiographic findings include valve fenestrations, nodular valve thickening and non-oscillating targets [3] (Table 1).

**Table 1:** Clinical criteria for the diagnosis of infective endocarditis [2, 3]

Major criteria	
Positive blood cultures for IE	Typical microorganisms consistent with IE from 2 separate blood cultures: • *Viridans streptococci*, *Streptococcus bovis*, HACEK group, *Staphylococcus aureus*; or • Community-acquired enterococci, in the absence of a primary focus; or Microorganisms consistent with IE from persistently positive blood cultures, defined as follows: • At least 2 positive cultures of blood samples drawn > 12 h apart; or • All of 3 or a majority of > 4 separate cultures of blood (with first and last sample drawn at least 1 h apart) Single positive blood culture for *Coxiella burnetii* or antiphase I IgG antibody titer > 1: 800
Positive echocardiographic findings of IE	Vegetation, perivalvular abscess, new dehiscence of a prosthetic valve
New valvular regurgitation (heart murmur)	

Minor criteria	
Predisposing heart condition or injection drug use	
Fever (> 38 °C; > 100.4°F)	
Vascular phenomena	Major arterial emboli, septic pulmonary infarcts, mycotic aneurysm, intracranial hemorrhage, conjunctival hemorrhages, Janeway lesions
Immunologic phenomena	Glomerulonephritis, Osler’s nodes, Roth spots, positive rheumatoid factor
Blood cultures non-compatible with major criteria	Positive blood culture but not meeting major criterion as noted previously or serologic evidence of active infection with organism consistent with IE
Minor echocardiographic criteria	Non-oscillating targets, new valvular fenestrations, nodular valve thickening

IE infective endocarditis; HACEK *Haemophilus species, Aggregatibacter species, Cardiobacterium hominis, Eikenella corrodens, and Kingella species*; The diagnosis of infective endocarditis (clinical definition) is definite when 2 major criteria, or 1 major criterion and 3 minor criteria, or 5 minor criteria are meet; IE possible when neither non-definite nor rejected-criteria are meet; IE rejected when a firm alternate diagnosis of endocarditis is meet, or resolution of symptoms is achieved with antibiotic therapy for 4 days or less [2, 3]

**Fig. 2:**
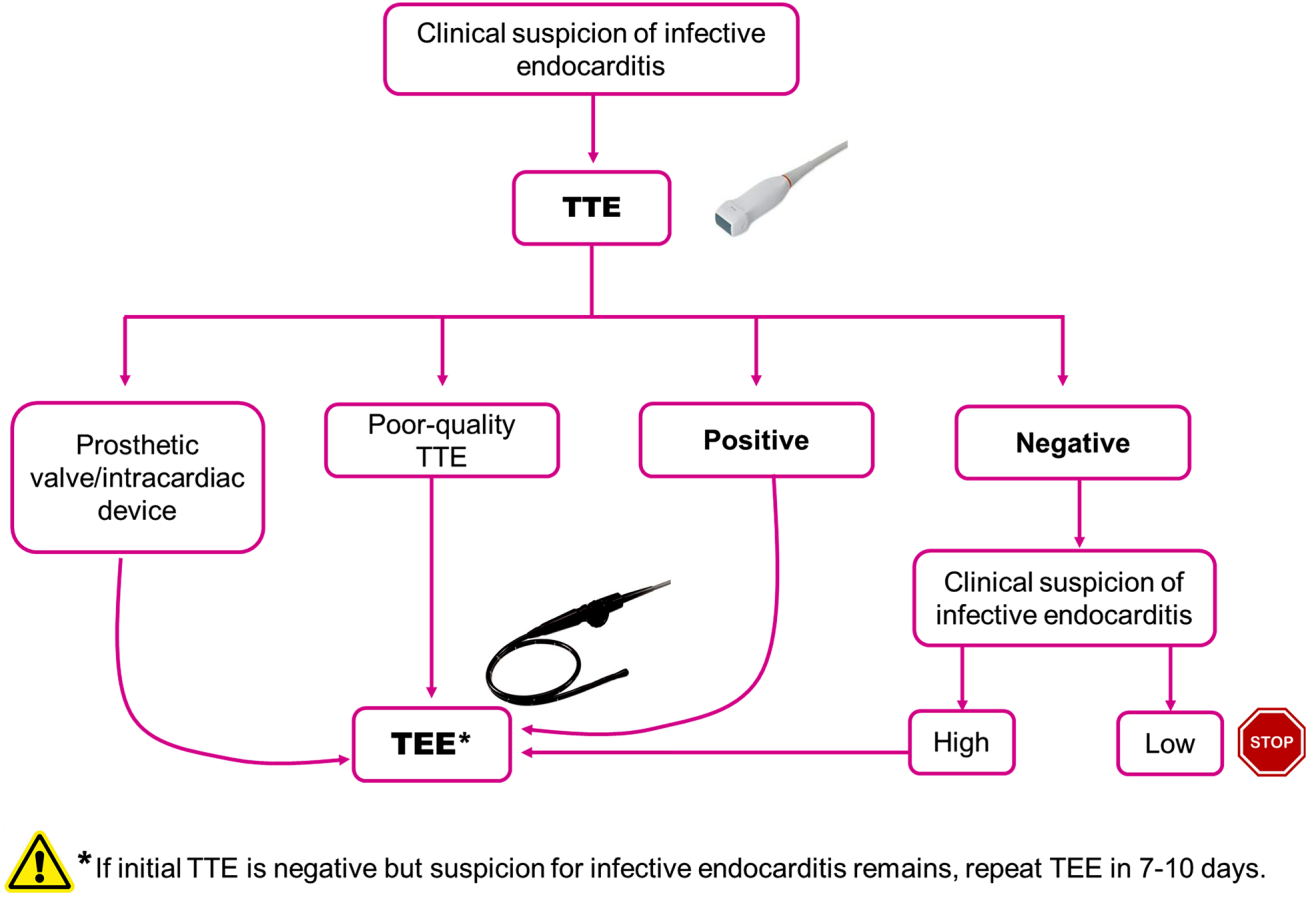
Diagnostic algorithm for echocardiographic diagnosis of infective endocarditis. *TTE* transthoracic echocardiogram, *TEE* transesophagic echocardiogram. Modified from [1]

In general, sensitivity of TTE is lower for native-valve endocarditis compared to transesophageal echocardiogram (TEE). TTE has a sensitivity of about 75% for detection of vegetations in native-valve IE, which rises to around 90% for TEE [1]. Specificity is high for both methods (about 90%) [1]. For perivalvular abscess, the sensitivity of TTE is about 50%, reaching about 90% for TEE. Specificity is also similar for both methods (about 90%). For prosthetic-valve endocarditis and device-related IE, TEE has a better sensitivity compared to TTE. Despite the high sensitivity and specificity of TEE, around 15% of patients show false-negative echocardiograms. Therefore, TEE should be repeated in 7–10 days if the suspicion of IE remains high. As the first method to use, TTE is non-invasive, aids in detecting vegetations and also provides crucial information about the systolic and diastolic function of both ventricles as well as the repercussion of valvular regurgitation on the cardiac function (i.e., low cardiac output, high left ventricular filling pressure/pulmonary edema). Limitations of TTE are a poor acoustic window, detecting small-sized vegetations and prosthetic valve/device-related endocarditis. While the initial exam to be performed is TTE in all patients with suspicion of IE, TEE should be always subsequently performed in most cases (Fig. 2). For right-sided endocarditis, TTE is not inferior to TEE [1].

Knowing the size of the vegetations aids in estimating the risk of embolism (> 10 mm high risk; > 15 mm very high risk) [1]. In general, vegetations in the mitral valve have a high risk of embolism, as observed in our patient, while IE of the aortic valve produce more often destructive lesions (abscess formation) [1]. The most important differential diagnosis of vegetations include tumors, thrombus, catheters/leads, papillary muscle or chordae tendineae rupture, prominent Eustachian valve and Chiari network, and also non-infective vegetations (marantic endocarditis) [1].

Surgery is typically indicated in native-valve endocarditis causing refractory pulmonary edema or shock, perivalvular abscess, large aortic or mitral vegetations (> 10 mm) following one or more embolic events, in prosthetic-valve endocarditis and in cardiac device-related IE.

## Conclusions

IE is an entity with high morbidity and mortality. Early detection and timely treatment are pivotal, for which point-of-care echocardiogram (TTE at first instance, and then TEE) has a critical role.

## Supplementary Information

**Additional file 1: Video S1 **Parasternal long axis (PLAX) view depicting a large vegetation attached to the atrial side of the anterior leaflet of the mitral valve.

**Additional file 2: Video S2** PLAX showing a severe mitral regurgitation triggered by the vegetation.

**Additional file 3: Video S3** Right ventricular inflow view (zoom view) showing a large vegetation in the atrial side of the posterior leaflet of the tricuspid valve.

## Data Availability

Not applicable.

## References

[1] Habib G, Badano L, Tribouilloy C, Vilacosta I, Zamorano JL, Galderisi M, Voigt JU, Sicari R, Cosyns B, Fox K, Aakhus S, European Association of Echocardiography (2010). Recommendations for the practice of echocardiography in infective endocarditis. Eur J Echocardiogr.

[2] Li JS, Sexton DJ, Mick N, Nettles R, Fowler VG, Ryan T, Bashore T, Corey GR (2000). Proposed modifications to the Duke criteria for the diagnosis of infective endocarditis. Clin Infect Dis.

[3] Durack DT, Lukes AS, Bright DK (1994). New criteria for diagnosis of infective endocarditis: utilization of specific echocardiographic findings duke endocarditis service. Am J Med.

[4] Philip M, Tessonier L, Mancini J, Mainardi JL, Fernandez-Gerlinger MP, Lussato D, Attias D, Cammilleri S, Weinmann P, Hagege A, Arregle F, Martel H, Oliver L, Camoin L, Casalta AC, Casalta JP, Gouriet F, Riberi A, Lepidi H, Raoult D, Drancourt M, Habib G (2020). Comparison between ESC and duke criteria for the diagnosis of prosthetic valve infective endocarditis. JACC Cardiovasc Imaging.

